# Therapeutic Potential of Exosomes in Pulmonary Fibrosis

**DOI:** 10.3389/fphar.2020.590972

**Published:** 2020-12-04

**Authors:** Linshen Xie, Ye Zeng

**Affiliations:** ^1^ West China School of Public Health and West China Fourth Hospital, Sichuan University, Chengdu, China; ^2^ Institute of Biomedical Engineering, West China School of Basic Medical Sciences and Forensic Medicine, Sichuan University, Chengdu, China

**Keywords:** exosome, epithelial mesenchymal transition, pulmonary fibrosis, bone marrow mesenchymal stem cell, type II alveolar epithelial cell, myofibroblast

## Abstract

Pulmonary fibrosis is closely associated with the recruitment of fibroblasts from capillary vessels with damaged endothelial cells, the epithelial mesenchymal transition (EMT) of type II alveolar epithelial cells, and the transformation of fibroblasts to myofibroblasts. Recent studies suggest that EMT is a key factor in the pathogenesis of pulmonary fibrosis, as the disruption of EMT-related effector molecules can inhibit the occurrence and development of PF. With the numerous advancements made in molecular biology in recent years, researchers have discovered that exosomes and their cargos, such as miRNAs, lncRNAs, and proteins, can promote or inhibit the EMT, modulate the transformation of fibroblasts into myofibroblasts, contribute to the proliferation of fibroblasts and promote immunoregulatory and mitochondrial damage during pulmonary fibrosis. Exosomes are key factors regulating the differentiation of bone marrow mesenchymal stem cells (BMSCs) into myofibroblasts. Interestingly, exosomes derived from BMSCs under pathological and physiological conditions may promote or inhibit the EMT of type II alveolar epithelial cells and the transformation of fibroblasts into myofibroblasts to regulate pulmonary fibrosis. Thus, exosomes may become a new direction in the study of drugs for the treatment of pulmonary fibrosis.

## Introduction

Pulmonary fibrosis (PF) is a pathological process that occurs after lung tissue damage and is the final manifestation of many lung diseases. PF has been shown to be a complex pulmonary interstitial disease caused by multiple factors, such as genetic abnormalities, autoimmunity, occupational exposure, trauma, radioactive element exposure, pathogenic microorganism infection, and drug administration (King et al., 2011; Meyer, 2017). Patients with PF typically have progressive dyspnea accompanied by irritating dry cough, with respiratory functions that will deteriorate with the aggravation of PF, eventually leading to respiratory failure and death (Thannickal et al., 2004). The median survival time of PF patients is only 3–5 years after diagnosis (Hopkins et al., 2016; Yanagihara et al., 2020). Diffuse pneumonia appears in the early stage of PF, while an excessive proliferation of fibroblasts and the deposition of extracellular matrix (ECM) appear during the later period (Gross and Hunninghake, 2001; Kinoshita and Goto, 2019).

Silicosis is an occupational disease with the highest morbidity and mortality in China and worldwide. Silicosis, which has a long incubation period and is difficult to detect, can still occur after an individual is no longer subject to silica dust exposure (Liu et al., 2013). From 1999–2018 in the USA, pneumoconiosis deaths among coal workers associated with pneumoconiosis and silicosis decreased by 69.6 and 53.0%, respectively (Bell and Mazurek, 2020). As reported in the Statistical Bulletin of Health Development in China at 2019, more than 19,428 cases of occupational diseases have been reported in China, of which more than 15,898 are cases of pneumoconiosis, with silicosis accounting for 81.8% of these cases. Silicosis is associated with a 61% overall increase in lung cancer risk (Liu et al., 2013) and is characterized by diffuse fibrosis of lung tissue, which seriously affects the quality of life survival time of patients (Barnes et al., 2019; Hoy and Chambers, 2020). Due to its unclear pathogenesis and a lack of clinically effective therapeutic drugs, the development of new methods and drugs for the prevention and treatment of PF has become a hot topic (Worthington and Hagood, 2020). Thus, there is an urgent need to identify effective intervention targets and develop methods and strategies to control and delay PF.

It is currently believed that the pathogenesis of PF can be divided into following three processes (Wynn, 2011; Kropski and Blackwell, 2019; Suryadevara et al., 2020): 1) susceptibility processes, including genetic mutations, environmental exposure, and aging processes, which make individuals susceptible to PF; 2) activation processes, including transforming growth factor-β (TGF-β) activation, fibroblast recruitment from capillary vessels with damaged endothelial cells, the epithelial mesenchymal transition (EMT), and the unfolded protein response (UPR) activation, which promote the progress of fibrosis.; and 3) progressive processes, including pathological fibroblast differentiation, endothelial cell death with vessel loss and new vessel formation, matrix deposition and remodeling, increased stiffness, and pro-fibrotic epigenetic changes in fibroblasts and epithelial cells. The EMT of damaged lung epithelial cells causes continuous mesenchymal cell activation and matrix remodeling, accelerating the production of excessive ECM deposition and the formation of fibrous scar tissue, which eventually leads to lung failure.

Recent studies have suggested that injury to type II alveolar epithelial cells, the interaction between alveolar epithelial cells and fibroblast, and the EMT play crucial roles in the pathogenesis of PF (Kropski and Blackwell, 2019; Yanagihara et al., 2020). EMT promotes fibrosis by activating signaling pathways associated with this process. The disruption of EMT-related effectors can inhibit the occurrence and development of PF (Kropski and Blackwell, 2019). With the continuous advancements in molecular biology in recent years, researchers have discovered that some regulatory microRNAs (miRNAs) and exosomes are closely associated EMT (Xu et al., 2019; Feng et al., 2020). Exosomes are present in various body fluids, such as blood, urine and sputum, and they can be used as potential biomarkers and treatments for different diseases, including PF (Keller et al., 2011; Guiot et al., 2019; Fukushima et al., 2020; Khalaj et al., 2020). The goal of this article is to review recent research progress on the role of EMT in the pathogenesis of PF and the potential role of exosomes and their specific cargoes.

### Exosomes and Epithelial Mesenchymal Transition

Exosomes are extracellular vesicles (EVs) that have become a hot topic in the field of EV research (Colombo et al., 2014; Cocozza et al., 2020). Exosomes are extracellular nanovesicles with a lipid bilayer structure that are released by cells through efflux and have diameters ranging from 30–150 nm (van Niel et al., 2018; Mathieu et al., 2019). Exosomes are nanoscale vesicles secreted by cells under physiological or pathological conditions (Wen et al., 2017; Stahl and Raposo, 2019). Exosomes are secreted from cells via exocytosis and absorbed by target cells, resulting in the transfer of biological signals (Yang et al., 2018). Many types of cells have been shown to secrete exosomes, such as B cells, epithelial cells, fibroblasts, and a variety of tumor cells (Mehryab et al., 2020; Moghiman et al., 2020; Peng et al., 2020). As important mediators of intercellular communication, the functions of exosomes are dependent on their cargo profile, which includes miRNAs, messenger RNAs, DNA, and proteins that contribute to both physiological functions and the pathophysiologies of a variety of diseases, including PF (Colombo et al., 2014; Akbari and Rezaie, 2020; Cocozza et al., 2020; Khalaj et al., 2020).

EMT is a process in which fully differentiated epithelial cells gradually transform into mesenchymal cells and lose their original function after exposure extracellular factors (Nieto et al., 2016). EMT is an important mechanism leading to the generation of myofibroblasts. During the EMT process, epithelial cells undergo significant changes in transcriptional regulation, cytoskeleton shape, adhesion, and ECM components (Singh et al., 2018). Under normal conditions, epithelial cells are tightly connected through mechanisms promoting adhesion between cells. During EMT, cells separate from neighboring cells and migrate to adjacent tissues, altering their function. During EMT, the expression of epithelial cell markers, such as E-cadherin, cytoplasmic zonula occludens protein 1 (ZO-1), and α-catenin are decreased, while that of fibroblast specific protein 1 (FSP-1), N-cadherin, vimentin, fibronectin and α-smooth muscle actin (SMA) are increased (Singh et al., 2018). Based on differences in biological environments, EMT can be divided into three subtypes (Nieto et al., 2016). Type I EMT is associated with embryonic development, including processes such as embryonic differentiation, organ formation, and nervous system differentiation. Type II EMT occurs during tissue repair, regeneration, and organ fibrosis. Under normal circumstances, type II EMT will cease when the inflammatory reaction is inhibited, but it will continue to aggregate fibroblasts if the injury and inflammation are not improved. Type III EMT is involved in tumor metastasis. During this process, the deformation, migration, and anti-apoptosis abilities of epithelial cells are enhanced and are associated with a lack of cell polarity, a weakening of cell adhesion, cytoskeletal remodeling, and the disappearance of other cell movement elements. Compared to type I and type III EMT, type II EMT is caused by persistent injury and inflammation, which is an important organ fibrosis process (Xu et al., 2019).

Exosomes are closely associated with EMT, a process that involves the disappearance of epithelial cell characteristics and the appearance of a mesenchymal cell phenotype and the ability to migrate (Singh et al., 2018). This is a complex process that involves a number of changes, such as cytoskeletal remodeling and reduced E-cadherin levels. Exosomes can promote or inhibit EMT, thereby contributing to the formation of the tumor microenvironment and various pathological processes, such as tumor metastasis, kidney injury, and fibrosis (Syn et al., 2016; Xiao et al., 2016; Conigliaro and Cicchini, 2018). Previous studies have shown that exosomes also play a role in immune regulation, the transition of epithelial cells into fibroblasts and mesenchymal cells (EMT), and the transformation of fibroblasts to myofibroblasts (Hu et al., 2020; Li et al., 2020b; Qin et al., 2020). Table 1 summarizes the exosomal cargos that promote or inhibit the EMT of lung epithelial cells.

**TABLE 1:** Exosomal cargos promote or inhibit the EMT of lung epithelial cells.

Exosomal cargo	Parental cells	Recipient cells	References
Inducer			
miR-210	Cancer-related fibroblasts	Non-small cell lung cancer cells (H1975 and A549)	(Yang et al., 2020)
Snail1	Cancer-related fibroblasts	Lung cancer cells	(You et al., 2019)
miR-499a-5p	A549, SPC-A-1-BM	A549, SPC-A-1-BM	(He et al., 2019b)
miR-200c, miR-205	Mesenchymal phenotype A549	N/A	(Tang et al., 2018)
*ZEB1* mRNA	Mesenchymal bronchial epithelial cells	Recipient epithelial cells	(Lobb et al., 2017)
miR-23a	TGF-β1-induced A549 cells	A549	(Kim et al., 2016)
miR-193a-3p, miR-210-3p, miR-5100	Hypoxic BMSCs	Lung epithelial cancer cells H358, A549, and H460	(Zhang et al., 2019)
N/A	Human umbilical cord MSCs	A549	(Zhao et al., 2018)
PD-L1	A549, H1299	A549, H1299 cells	(Hong et al., 2020)
miR-21	Bronchial epithelial cells (BEAS-2B)	Bronchial epithelial cells (BEAS-2B)	(He et al., 2019a)
Suppressor			
miR-26a	Human airway epithelial cell line 16HBE	—	(Tang et al., 2018)

### Exosomal Cargos and Epithelial Mesenchymal Transition Promotion

Exosomes exert their functions through the cargos they harbor and can carry molecules that promote EMT and act directly on target cells to mediate EMT. Exosomes contain a variety of molecules that can regulate cell function and promote EMT, such as matrix metalloproteinases (MMPs), hypoxia inducible factor-1α (HIF-1α), and so on (Hu et al., 2020; Kim et al., 2016). MiRNAs are major components of exosomes (Zeng et al., 2019). These molecules can bind to and regulate target mRNAs by inhibiting their translation or promoting their degradation and are widely involved in cell proliferation, apoptosis, development, differentiation, angiogenesis, and fibrosis (Kadota et al., 2020; Yao et al., 2020). Exosomes secreted by cancer-related fibroblasts and harboring miR-21, mir-378e and miR-143 can induce EMT in different breast cancer cell lines, and miR-21 can also promote EMT in renal tubular epithelial cells (Donnarumma et al., 2017). MiR-210 in exosomes derived from cancer-associated fibroblasts can induce the EMT of non-small cell lung cancer cells (H1975 and A549) via the PTEN/PI_3_K/AKT pathway (Yang et al., 2020). The level of SNAI1 in exosomes derived from cancer-related fibroblasts is associated with the level of SNAI1 in cancer-related fibroblasts, which is crucial for inducting EMT in lung cancer cells (You et al., 2019). The level of exosomal miR-499a-5p is associated with the level of miR-499a-5p and the EMT of the human lung cancer and human lung adenocarcinoma cell lines A549 SPC-A-1-BM (He et al., 2019b). MiR-23b-enriched exosomes from TGF-β1-induced A549 cells can promote the EMT of A549 (Kim et al., 2016), while miR-23a can promote TGF-β1-induced EMT in lung cancer cells (Cao et al., 2012). Exosomes derived from mesenchymal bronchial epithelial cells but not epithelial bronchial epithelial cells contain *ZEB1* mRNA and can induce the EMT of recipient epithelial cells (Lobb et al., 2017). MiR-200c and miR-205 levels in exosomes from invasive cells (mesenchymal phenotype A549) are significantly decreased compared to those observed in noninvasive cells (16HBE cells) (Tang et al., 2018), with both miR-200c and miR-205 being EMT inducers (Tellez et al., 2011).

EMT induced by exosomal cargos is associated with immune regulation. The knockdown of circular RNA (cirRNA) cir-CPA4 can inhibit the growth, mobility and EMT of A549 cells by inhibiting exosomal PD-L1 levels (Hong et al., 2020). The exposure of macrophages to apoptotic cells inhibits TGF-β1-induced EMT in primary mouse lung alveolar epithelial cells *in vivo* and *in vitro* (Yoon et al., 2016). The results of a recent study further suggest that conditioned medium from macrophages exposed to apoptotic cancer cells inhibits the TGF-β1-induced EMT of A549 cells (Kim et al., 2019). Cigarette smoke extract was shown to induce the production of EVs with low miR-21 levels from bronchial epithelial cells to ameliorate EMT in chronic obstructive pulmonary disease by alleviating M2 macrophage polarization (He et al., 2019a). Reversing the expression of EMT mediators in exosomes or suppressing the release of EMT-related exosomes would be a viable therapy for lung fibrosis. A recent study showed that aspirin reversed the EMT of nasopharyngeal carcinoma cells by promoting miR-203 expression and repressed exosomal latent membrane protein 1 (LMP1) secretion in Epstein-Barr virus-positive cells (Zuo et al., 2019). Exosomal LMP1 promotes the EMT potential of Epstein-Barr virus-negative cells.

### Exosomal Cargos and Epithelial Mesenchymal Transition Inhibition

Exosomes can also inhibit EMT through their cargos. Exosomal miR-1255b-5p can suppress the EMT of colorectal cancer cells by inhibiting Wnt/β-catenin activation (Zhang et al., 2020), while exosomal miR-34c from mesenchymal stem cells can inhibit the EMT of nasopharyngeal carcinoma cells by targeting β-catenin (Wan et al., 2020). In another study, MSC-derived exosomal miR-3940-5p was shown to inhibit the EMT of colorectal cancer by targeting integrin alpha6 (ITGA6) (Li et al., 2020d), while exosomal miRNA-215-5p in adipose-derived stem cells inhibited the EMT of podocytes by inhibiting ZEB2 (Jin et al., 2020). MiR-26a levels in exosomes from invasive cells (mesenchymal phenotype A549) are significantly increased compared to those in exosomes from noninvasive cells (16HBE cells) (Tang et al., 2018). MiR-26a is an EMT suppressor (Liang et al., 2014). However, the results of recent studies have indicated that EMT-inhibiting exosomal cargos are almost miRNAs, and whether exosomal proteins, lipids, and even long non-coding RNA (lncRNAs) can inhibit EMT, and their associated mechanisms, remain to be explored in depth.

### Regulation of Epithelial Mesenchymal Transition by Exosomes Derived From Mesenchymal Stem Cells

Exosomes derived from mesenchymal stem cells (MSCs) has been shown to regulate EMT. Exosomal miRNAs, including miR-193a-3p, miR-210-3p and miR-5100 derived from hypoxic bone marrow mesenchymal stem cells (BMSCs) activate STAT3 signaling and enhance the EMT of lung epithelial cancer cells (Zhang et al., 2019). Human umbilical cord MSCs promote the EMT, invasion, and migration of A549 lung cancer cells via MSC-derived exosomes, while silencing TGF-β1 expression in MSCs can inhibit the EMT-promoting effect of MSCs on A549 cells via MSC-derived exosomes (Zhao et al., 2018). However, human umbilical cord MSC-exosomal miR-3940-5p inhibits the invasion and EMT of colorectal cancer cells (Li et al., 2020d). Exosomes derived from human umbilical cord MSCs can suppress the EMT and liver fibrosis (Li et al., 2013). Therefore, the effects of exosomes derived from different MSCs on EMT is dependent on the source of MSCs under specific conditions, and the molecules in MSC-derived exosomes associated with EMT remain unclear.

### Primary Epithelial Mesenchymal Transition Signaling Pathways

#### Wnt Signaling Pathway

The Wnt signaling pathway comprises three primary pathways: the Wnt/β-catenin, Wnt/JNK, and Ca^2+^-mediated Wnt/Ca^2+^ signaling pathways (Kohn and Moon, 2005; Teeuwssen and Fodde, 2019). The results of a literature search revealed 19 types of Wnt genes in the human genome that encode functionally distinct proteins (Wodarz and Nusse, 1998). Among them, Wnt2, Wnt3, and Wnt8b are involved in classic Wnt/β-catenin signaling pathways, while Wnt6, Wnt7b, and Wnt10a are involved in nonclassical Wnt signaling pathways, including the Wnt/JNK and Ca^2+^-mediated Wnt/Ca^2+^ pathways. The classic Wnt/β-catenin signaling pathway is an important pathway that regulates the occurrence and development of EMT (Teeuwssen and Fodde, 2019) and comprises the secreted protein Wnt family, secreted frizzled-related protein (sFRP), protein phosphatase 2A (PP2A), transmembrane receptor frizzled protein Fz, casein kinase (CK1), the Dickkopf protein (DKK) family, glycogen synthase kinase-3β (Gsk-3β), β-catenin, and adenomatous polyposis coli (APC).

When Wnt is activated, the extracellular domain of the Wnt receptor Fz binds to Wnt, after which β-catenin cannot be degraded in the cytoplasm. β-Catenin functions as a carrier to mediate the formation of the E-cadherin and α-catenin complex, inhibiting the activation of E-cadherin. After accumulating to a specific level, β-catenin is translocated to form a complex with nuclear transcription factor (TCF/LEF) to activate downstream target genes such as snail and slug, thereby promoting EMT (Teeuwssen and Fodde, 2019). After Wnt signal activation, GSK-3β activity is inhibited, which decreases the degradation of β-catenin, inducing cell morphology changes and regulating the EMT. The Wnt/β-catenin signaling-mediated induction of EMT is involved in various cancers, such as prostate cancer and ovarian cancer (Teeuwssen and Fodde, 2019; Yeh et al., 2019), as well as organ fibrosis such as renal fibrosis and liver fibrosis (Edeling et al., 2016; Wang et al., 2018a).

Exosomes released from colon cancer cells with high levels of the Wnt receptor frizzled 10 protein (FZD10) can stimulate EMT activation to reprogram normal colonic epithelial cells (Scavo et al., 2020). Cancer stem cell-like cell-derived exosomal lncRNA DOCK9 antisense RNA2 (AS2) can activate the Wnt/β-catenin pathway to induce EMT and promote the progression of papillary thyroid carcinoma (Dai et al., 2020). LncRNA ADAMTS9-AS1 can suppress the EMT of colorectal cancer cells and is markedly reduced in serum exosomes from colorectal cancer patients (Li et al., 2020c). Therefore, exosomes can regulate the EMT via the Wnt signaling pathway. However, how Wnt/β-catenin signaling regulates exosomes and contributes to PF remains to be investigated.

#### Rho-ROCK Signaling Pathway

The Rho-ROCK signaling pathway is commonly used by various types of cells (Johan and Samuel, 2019). Rho family proteins have guanosine triphosphate (GTP) enzyme activity and are also known as RhoGTPases, members of which belongs to the Ras superfamily of GTP binding proteins. There are more than 20 different Rho proteins in mammalian cells. Rho family proteins are believed to regulate multiple biological processes including cytoskeleton remodeling, cell morphology changes, cell proliferation, and adhesion through intracellular signaling pathways (Porazinski et al., 2020). Rho-associated coil forming protein kinase (ROCK) is the downstream target effector of Rho. ROCK receives the activation signal transmitted by Rho, which promotes the phosphorylation of its amino acid domain and leads to a series of phosphorylation and dephosphorylation reactions. Rho-ROCK signaling is a key regulator of actomyosin-mediated contractility, cell shape, and cytoskeletal arrangement, thereby affecting cell functions such as cell proliferation, differentiation, motility, and adhesion. The inhibition of Rho/ROCK signaling can downregulate the expression of EMT-related molecules (Liu et al., 2020). The primary proximal tubule cells in a hypertensive chronic kidney disease model showed upregulation of Rho-ROCK signaling accompanied by EMT, which was characterized by the development of spindle shape morphology, gene expression changes in EMT markers (collagen type I alpha 3, MMP9, BMP7, and occludin) and increased N-cadherin and vimentin expression (Jia et al., 2015). Incubating human bronchial epithelial cells (BECs) with silica *in vitro* induced the *de novo* expression of α-SMA and vimentin and decreased that of E-cadherin. In addition, silica treatment of human BECs could result in Rho activation. In another study, an inhibitor of a Rho effector protein could upregulate the E-cadherin expression while attenuating α-SMA and vimentin expression in silica-stimulated cells. All of these results demonstrated that activation of the Rho-ROCK signaling pathway is most likely involved in silica-induced EMT in human BECs (Hu et al., 2013).

Liver fibrosis has been shown to be reduced by the administration of the ROCK1 inhibitor fasudil, which was accompanied by reduced serum levels of EVs (Hirsova et al., 2016). However, the link between the Rho-ROCK signaling pathway and exosomes in PF remains poorly studied.

#### Transforming Growth Factor-β/Smad Pathway

TGF-β1 is considered to be the most important factor in PF. TGF-β1 not only induces alveolar EMT but also promotes the differentiation of bone marrow mesenchymal stem cells (BMSCs) into myofibroblasts (Li et al., 2020a; Qian et al., 2020; Yang et al., 2012). TGF-β1 levels were shown to be significantly reduced during the BMSC-conditioned medium-mediated inhibition of silicosis (Wang et al., 2018b). After the body is stimulated by inflammatory or injury factors, such as endotoxin and reactive oxygen species, a variety of cells (including type II alveolar epithelial cells) produce TGF-β1 (Kumari et al., 2017).

The TGF-β superfamily comprises 25 proteins, including TGF-βs, activins, inhibins, and growth differentiation factors (GDFs). GDFs play an important role during embryonic development, cell division, organ formation, and tissue repair. TGF-β1 is one of the most important TGF-β subtypes and can be secreted by endothelial cells, T lymphocytes, B lymphocytes, and fibroblasts and is expressed in various organs and tissues (Hayashi and Sakai, 2012; Lichtman et al., 2016). It plays a role in cell division, proliferation, differentiation, apoptosis, and migration. TGF-β1 stimulates the production of ECM and promotes the degradation of ECM. TGF-β receptors (TβRs) are widely expressed in various tissues. There are three subtypes of TGF-β, including TGF-β1, TGF-β2, and TGF-β3, among which β1 and β2 are signal transduction receptors with structures comprising extracellular, transmembrane, and intramembrane regions (Budi et al., 2017; Kang et al., 2009). The extracellular domain of TβR is rich in cysteine, which can bind to the ligand and activate the downstream signal cascade. TβRI mediates the nuclear localization of Smad3, the induction of α-SMA during the collagen-induced EMT of alveolar epithelial cells, and fibroblast accumulation during idiopathic pulmonary fibrosis (IPF) (DeMaio et al., 2012). TβRII knockout can inhibit the normal nuclear localization of β-catenin, causing cell death, cell cycle arrest, increased of fibrotic factor expression, and renal fibrosis (Nlandu-Khodo et al., 2017). TβRII knockdown can also promote PF, while hydroxysafflor yellow A can inhibit the TGF-β1-mediated activation of fibrotic changes by targeting TβRII in human fetal lung fibroblasts (MRC-5) (Pan et al., 2017).

TGF-β1 signaling pathways primarily comprise the TGF-β1/Smad signaling pathway and the Smad-independent signaling pathway (Shi and Massagué, 2003; Macias et al., 2015). Smad is a transcriptional coordination factor of the TGF-β superfamily present in the cytoplasm and mediates TGF-β signaling in the nucleus. At present, 9 Smad proteins have been identified that are divided into three subtypes (Sun et al., 2014; Ng-Blichfeldt et al., 2019). Membrane receptor-regulated Smads (R-Smads) include Smad1, Smad2, Smad3, Smad5, and Smad8, among which Smad2 and Smad3 bind to TGF-β1 to form a transcription complex that binds to a specific region of the downstream target gene and then participates in TGF-β signal transduction. Common pathway Smads (co-Smads) include Smad4 and Smad10, where Smad4 binds R-Smad to form a heterologous complex and enter the nucleus to participate in transcription al regulation. Inhibitory Smads (I-Smads) include Smad 6 and 7, which play a negative role in regulating the TGF-β1 signal transduction pathway. The TGF-β signaling pathways include the classical and nonclassical Smad signaling pathways. The classical Smad signaling pathway is the primary pathway involved in the TGF-β-mediated induction of fibrosis. The Smad-independent signaling pathway primarily involves the mitogen-activated protein kinase (MAPK) pathway (Du et al., 2020; Luo et al., 2020), which includes three members: p38MAPK, extracellular signal-related kinase (ERK), and c-Jun N-terminal kinase (JNK). Cross-talk has been shown to occur between the TGF-β1 and Wnt/β-catenin signaling pathways (Basile and Mehrotra, 2017).

TGF-β1 directly activates Smad signaling, which triggers pro-fibrotic gene overexpression. TGF-β1-induced EMT had been widely demonstrated in different pulmonary diseases, such as asthma (Ijaz et al., 2014), pulmonary cystic fibrosis (Rout-Pitt et al., 2018), IPF and hypersensitivity pneumonitis (Kiszalkiewicz et al., 2017). In Smad3-deficient mouse models, type I procollagen expression is suppressed, and the hydroxyproline content in the lungs is reduced compared to wild-type mice treated with bleomycin (Zhao et al., 2002). Furthermore, the loss of Smad3 greatly attenuates the morphological fibrotic response to bleomycin in the mouse lung. These results demonstrate that Smad3 contributes to bleomycin-induced lung injury and that Smad3 may serve as a novel target for the potential treatment of lung fibrosis (Zhao et al., 2002).

The exosomal delivery of TGF-β contributes to the activation of TGF-β signaling under pathological conditions such as tumor and hypoxia, contributing to EMT and myofibroblast differentiation (Ahuja et al., 2020; Chowdhury et al., 2015). Exosomes derived from human umbilical cord MSCs can reduce TGF-β levels and liver fibrosis *in vivo* and inhibit EMT *in vitro* (Li et al., 2013). Similarly, silencing TGF-β1 expression in human umbilical cord MSCs can revert the EMT-promoting effect of MSCs on A549 cells via MSC derived exosomes (Zhao et al., 2018). BMSC-derived exosomes can reverse EMT in rabbit endometrial epithelial cells induced by TGF-β1, inhibiting endometrial fibrosis (Yao et al., 2019b). Thus, the differences between BMSC-derived exosomes from cells under normal and abnormal conditions such as cancer, hypoxia, and pro-fibrosis should not be neglected.

**FIGURE 1:**
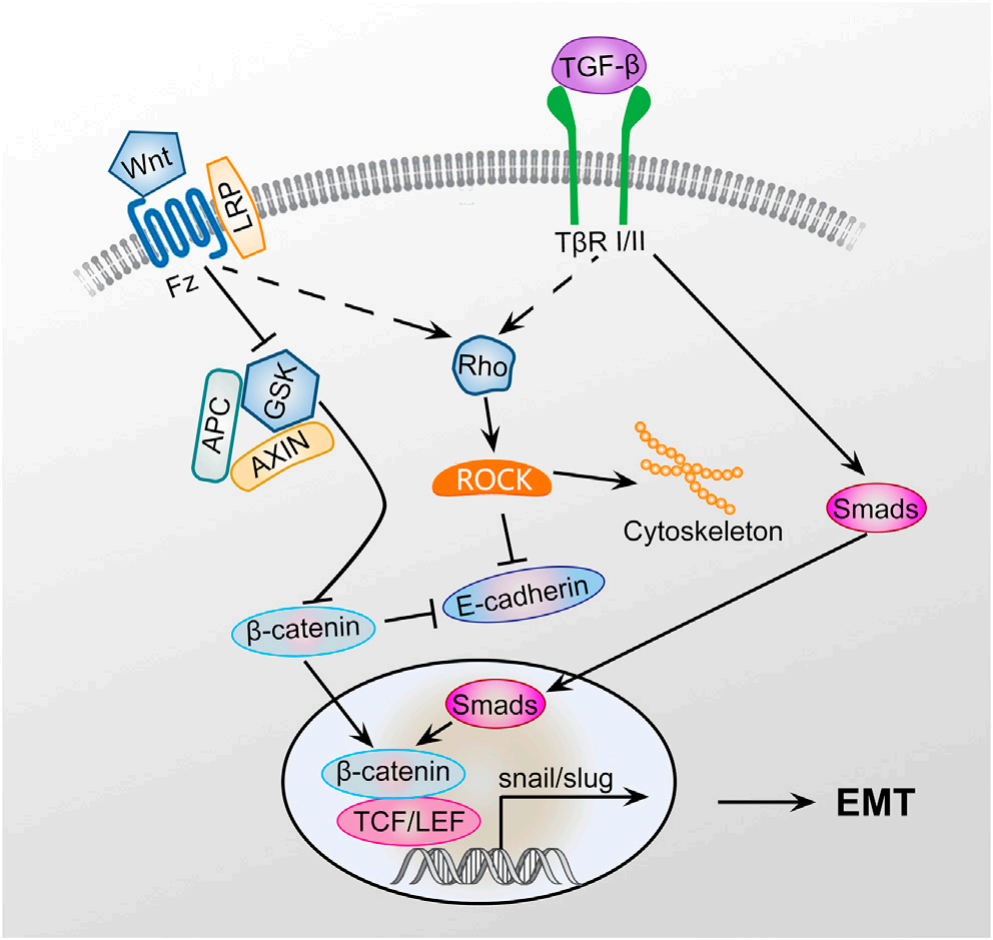
Signaling pathway of EMT. GSK3β is an important negative regulator of the Wnt signaling pathway. Axin can interact with many important members of the Wnt signaling pathway to negatively regulating Wnt/β-catenin signaling. Wnt signaling pathway activation inhibits the phosphorylation of GSK3β, preventing GSK3β from phosphorylating β-catenin, leading to the inhibition of β-catenin degradation. GSK3β phosphorylation can promote axin autophosphorylation, increasing the affinity of axin and β-catenin and promoting β-catenin degradation. β-Catenin can promote the EMT of type II alveolar epithelial cells, promote the transformation of type II alveolar epithelial cells to myofibroblasts, and ultimately promote PF by activating the TGF-β1/Smad and Rho-ROCK signaling pathways.

EMT is a key step in development, wound healing, fibrosis, and cancer that involves a complex network of signaling pathways, including the TGF-β1/Smad, Rho-ROCK, and Wnt/β-catenin pathways (Figure 1). These signal factors can interact with each other through negative or positive feedback loops that forms a closed network (Zhang et al., 2016). EMT induction by exosomes with particulate matter having a diameter of less than 2.5 (PM_2.5_) may be associated with activation of the TGF-β/Smad and Wnt/β-catenin pathways to promote fibrogenesis (Xu et al., 2019). However, the detailed signaling mechanisms involved in exosomal cargo-mediated EMT and their contributions in PF remain unclear.

### Exosomes and Transformation of Fibroblasts to Myofibroblasts

EVs, especially exosomes, can regulate the differentiation of fibroblasts into myofibroblasts (Table 2). EV-associated miR-210 from primary human bronchial epithelial cells and lung fibroblasts induced by cigarette smoke extract acts as a paracrine autophagy mediator of myofibroblast differentiation in lung fibroblasts (Fujita et al., 2015). Activated primary human lung fibroblasts can release prostaglandins such as PGE_2_-enriched exosomes or EVs to inhibit TGF-β1-induced myofibroblast differentiation (Lacy et al., 2019), while miR-22 negatively regulates myofibroblast differentiation from lung fibroblasts and ameliorates PF in mice (Kuse et al., 2020).

**TABLE 2:** Regulation of lung fibroblast transdifferentiation by exosomes.

Exosomal cargo	Parental cells	Effect	References
miR-210 (EV)	Cigarette smoke extract-induced human bronchial epithelial cells and lung fibroblasts	Promotion	(Fujita et al., 2015)
PGE_2_	Human lung fibroblast	Inhibition	(Lacy et al., 2019
miR-22	PF mice	Inhibition	(Kuse et al., 2020)
WNT5A	Bronchoalveolar lavage (BAL) fluid	Promotion	(Martin-Medina et al., 2018)
MiR-125a-5p	Silica-exposed macrophages	Promotion	(Wang et al., 2020)
Let-7 and miR-99 families	Lung spheroid cell	Inhibition	(Dinh et al., 2020)

The number of EVs, especially exosomes, is increased in bronchoalveolar lavage (BAL) fluid from experimental lung fibrosis and patients with IPF and are rich in WNT5A (Martin-Medina et al., 2018). Interestingly, EV-associated WNT5A may be associated with fibroblast activation (Martin-Medina et al., 2018). A recent study showed that macrophages are the major secretors of exosomes containing early secreted pro-inflammatory cytokines in BAL fluid (Ye et al., 2020). Exosomal miR-125a-5p derived from silica-exposed macrophages induces the fibroblast to myofibroblast transition and silicosis (Wang et al., 2020). Exosomes derived from lung spheroid can could attenuate and resolve bleomycin- and silica-induced fibrosis (Dinh et al., 2020) and contain miRNAs in the let-7 and miR-99 families as well as numerous growth factors and ECM-related proteins. The identification of crucial molecules in exosomes may benefit the development of effective IPF therapies using secreted EVs (Dinh et al., 2020).

### Novel Mechanisms of PF Mediated by Exosomes

In addition to the EMT and transformation of fibroblasts into myofibroblasts, other mechanisms exist with respect to the modulation of PF by exosomes. MiR-328 derived from M2 macrophages promotes lung fibroblast proliferation and PF (Yao et al., 2019a). In another study, exosomal miR-142-3p from macrophages was shown to attenuate PF, possibly by reducing the expression of TGFβI and pro-fibrotic genes in alveolar epithelial cells and lung fibroblasts (Guiot et al., 2020). BMSCs promote an immunoregulatory, anti-inflammatory monocyte phenotype to prevent PF (Mansouri et al., 2019). Exosomal let-7 from menstrual blood-derived endometrial stem cells alleviates lung fibrosis and alveolar epithelial cell damage in mice by regulating reactive oxygen species levels, mitochondrial DNA damage and NLRP3 inflammasome activation (Sun et al., 2019). Lung fibroblast-derived EVs from IPF patients were shown to increase mitochondrial reactive oxygen species levels and associated mitochondrial damage in lung epithelial cells, and these EVs were sown to contain elevated levels of miR-23b-3p and miR-494-3p (Kadota et al., 2020). Thus, the proliferation of fibroblasts, immunoregulatory pathways, and mitochondrial damage are novel mechanisms and promising targets for exosomal PF therapy.

### Therapeutic Potential of BMSCs and Their Exosomes

The primary manifestation of PF is an abnormal increase in the number of interstitial cells and ECM levels (Worthington and Hagood, 2020). Myofibroblasts are the primary effector cells that synthesize and secrete ECM during PF (Kropski and Blackwell, 2019; Suryadevara et al., 2020; Wynn, 2011). In IPF, the myofibroblasts in fibrosis lesions are primarily derived from the transformation of the original fibroblasts in lung tissue, the migration and differentiation of bone marrow-derived fibroblasts in peripheral circulation, and the EMT of local alveolar epithelial cells (Worthington and Hagood, 2020). In renal fibrosis, 35% of myofibroblasts are derived from bone marrow, which depends on the TGF-β1 signaling pathway (LeBleu et al., 2013). BMSCs migrate to the injured liver and differentiate to myofibroblasts via cannabinoid receptor 1 (Wang et al., 2017). Nicotine exposure drives the differentiation of BMSCs into myofibroblasts in the lungs (Sakurai et al., 2018). However, autologous BMSCs treated with hepatocyte growth factor can effectively improve the adverse symptoms of silicosis patients and promote the absorption of silicosis nodules (Liu et al., 2015; Yan et al., 2016). The administration of normal BMSCs to mice with silicosis via tail vein injection can also reduce silicosis (Wang et al., 2018b). In one study, exosomes derived from Wharton’s jelly mesenchymal stem/stromal cells (WJMSCs) and BMSCs can modulate lung macrophage phenotypes, restore the lung architecture and decrease fibrosis in hyperoxia (75% O_2_)-exposed mice, while exosomes derived from human dermal fibroblasts could not (Willis et al., 2018). In another study, BMSCs overexpressing transforming growth factor-beta 3 (TGF-β3), an inhibitor of myofibroblast proliferation, could promote wound healing and reduce scar tissue formation in a rabbit model (Li et al., 2018a). In addition, the transplantation of BMSCs overexpressing miR-124a to diabetic nephropathy rats could mitigate the EMT of podocytes and renal fibrosis (Cai et al., 2020). Granulocyte-colony stimulating factor (G-CSF) can enhance the homing of autologous BMSCs to damaged lung tissue to inhibit bleomycin-induced PF (Zhao et al., 2020). Thus, BMSCs act as a double-edged sword, where BMSCs in PF patients serve as an important source of myofibroblasts that promote PF, while those in healthy subjects or under modified conditions may inhibit PF. Therefore, it is of great importance to determine the functional differences between BMSCs from normal physiological conditions and those from abnormal pathological conditions. The correct guidance and use of BMSCs could improve health and promote their further use in clinical practices.

Research on BMSCs has a long history. It was expected that BMSCs would migrate to damaged organs and exert similar regenerative and differentiation effects as pluripotent stem cells to promote organ repair (Luo et al., 2019; Tang et al., 2020). However, the implantation rate of BMSCs has been shown to be extremely low in animal models, while their culture supernatant can also promote the repair of injured organs (Lai et al., 2010). Therefore, researchers have begun to focus on the paracrine mechanism of BMSCs. Recent studies have shown that cell-cell information exchange may occur through membrane EVs that are rich in bioactive factors, with exosomes, an EV subtype, have been thoroughly studied (Lai et al., 2010; Roy et al., 2018). It is currently believed that exosomes derived from BMSCs not only have a similar efficacy as BMSC transplantation but also reduce the risks associated with BMSC transplantation, such as immune rejection, teratogenic tumors, and pulmonary embolism (Lai et al., 2010; Mehryab et al., 2020; Peng et al., 2020; Qin et al., 2020). Thus, studies on the use of exosomes derived from BMSCs to repair the damaged organs are increasing.

Studies have reported that one week after BMSCs were used to treat myocardial ischemia and reperfusion injury, less than 1% of BMSCs survived and did not differentiate into cardiomyocytes, but they protected the heart through the paracrine mechanism mediated by exosomes (Lai et al., 2010). Exosomes derived from human umbilical cord MSCs can effectively alleviate liver fibrosis (Li et al., 2013). Exosomes show exciting therapeutic effects in lung injury and PF, showing similar or even better therapeutic potential as MSCs. BMSC conditioned medium can improve alveolar epithelial wound repair, inhibit alveolar epithelial mesenchymal transition, and significantly inhibit silicosis (Choi et al., 2014; Li et al., 2018b; Wang et al., 2018b). Mouse adipose-derived MSCs and their exosomes can improve PF and inflammation in advanced silicosis models, and high concentration of exosomes can achieve therapeutic effects that are comparable to MSCs (Bandeira et al., 2018). In a model of silicosis, BMSCs can migrate to damaged lung tissue, but cannot differentiate into alveolar epithelial cells. Instead, they promote the proliferation of type II alveolar epithelial cells, inhibit type II alveolar epithelial fibrosis, and inhibit collagen deposition through the paracrine pathway (Hostettler et al., 2017; Li et al., 2017; Wang et al., 2018b). Therefore, BMSCs have limited ability to self-differentiate; rather they primarily act through paracrine exosomes. However, the molecules and signaling pathways through which exosomes regulate silicosis remain unclear.

**FIGURE 2:**
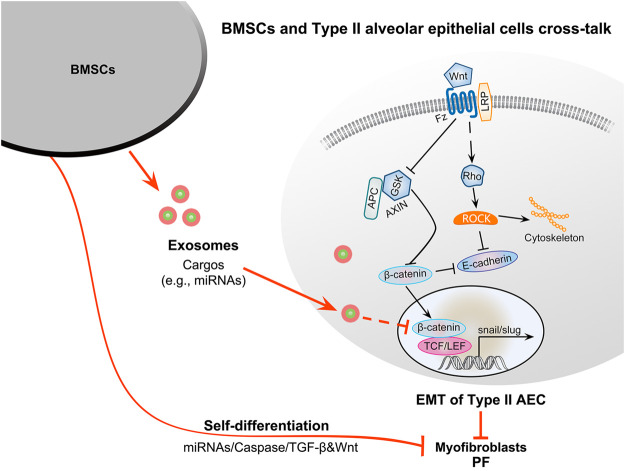
Therapeutic potential of BMSC-derived exosomes for PF and the potential molecular mechanisms. BMSCs in the PF microenvironment can contribute to myofibroblasts formation through self-differentiation and secrete exosomes to facilitate the EMT of type II alveolar epithelial cells to promote PF. In contrast, BMSCs from healthy individuals can migrate to damaged organs to promote their repair and secrete exosomes to inhibit the EMT of type II alveolar epithelial cells via the interplay between the Wnt and TGF-β1/Smad signaling pathways. Exosomal cargos such as miRNAs can inhibit β-catenin and axin activity. Axin can negatively regulate the Wnt/β-catenin signaling pathway, inhibiting the phosphorylation of GSK3β and leading to the suppression of β-catenin degradation. β-Catenin can promote the transformation of type II alveolar epithelial cells into myofibroblasts via EMT. Finally, the downregulation of axin can inhibit the EMT of type II alveolar epithelial cells to inhibit PF. The self-differentiation of BMSCs may be mediated by miRNAs, caspase, TGF-β1, and Wnt signaling pathways.

Exosomes are the key factor regulating the differentiation of BMSCs into myofibroblasts. Exosomes from normal BMSCs may suppresses EMT by interacting with the Wnt/β-catenin pathway in alveolar epithelial cells to inhibit PF (Figure 2).

## Summary

The occurrence of PF is closely associated with exosomes and the transition of epithelial cells into fibroblasts and mesenchymal cells (EMT), the transformation of fibroblasts, fibroblast proliferation, immunoregulatory effects, and mitochondrial damage. On the one hand, exosomes secreted by cells can affect the pathological processes of diseases by promoting the occurrence of EMT via their cargos, in which the microRNA contents are particularly relevant for the pathology of PF. For example, during the occurrence and development of PF, exosomes can upregulate the EMT and enhance the differentiation of BMSCs into myofibroblasts. Therefore, a more in-depth study on the role of exosomes in fibroblast proliferation, fibroblast activation, and myofibroblast differentiation can provide new insights into PF diagnosis and treatment. On the other hand, future studies may identify a large number of exosomal molecules that can promote fibrosis inhibition, EMT inhibition, and even reverse the EMT process, which would have anti-fibrotic effects and alleviate the functional damage of fibrosis to human tissues and organs, since evidence supports their potential for altering gene programs and inducing the differentiation or dedifferentiation of target cells. Therefore, further research on the effects of exosomal cargos in the process of tissue fibrosis may provide a new method for the treatment of fibrosis-related diseases. On the whole, with the continuous development of exosomal-related technologies, such as the isolation, identification, and modification of exosomes, exosomes may become a new direction in the research of drugs for the treatment of PF.

BMSCs have strong plasticity that acts as a double-edged sword. They can promote and antagonize fibrosis associated with the microenvironment changes. The differentiation and regulation of BMSCs is closed associated with exosomes. Exosomes with enriched specific cargos such as miR-214-3p may be used to treat silicosis by regulating the function of BMSCs and type II alveolar epithelial cells. Importantly, although BMSC-derived exosomes have a number of functions, such as in repairing tissue damage and suppressing inflammatory responses, the associated mechanisms are not fully understood, and the results remain controversial.

Most of the effects of exosomes observed in recent studies are from *in vitro* experiments, and only few cargos were identified, which should be investigated and validated *in vivo* in the future. Further *in vivo* and *in vitro* experimental studies to explore the molecular mechanism of BMSCs in the treatment of PF, to elucidate the therapeutic effects of BMSC-derived exosomes on the proliferation of type II alveolar epithelial cells and PF, and to reveal the regulatory mechanisms associated with TβR1 signaling and the Wnt/β-catenin pathway will provide a theoretical basis or new strategies for effectively terminating or reversing PF.

## Author Contributions

YZ contributed to the conception and design of the work. All authors drafted the manuscript and approved the final manuscript.

## Funding

Funding was provided by the National Natural Science Foundation of China (Grant nos. 11932014 and 11402153).

## Conflict of Interest

The authors declare that the research was conducted in the absence of any commercial or financial relationships that could be construed as a potential conflict of interest.
